# Supplementary material for: Ants Can Anticipate the Following Quantity in an Arithmetic Sequence
Source: Behav Sci (Basel). 2021 Jan 28;11(2):18. doi: 10.3390/bs11020018 (PMC7911458; doi:10.3390/bs11020018)
Supplement: Supplementary file 1 [file behavsci-11-00018-s001.pdf]

Supplementary Materials

Figure S1 illustrates the Experiment I, Figure S2 illustrates the Experiment 2.

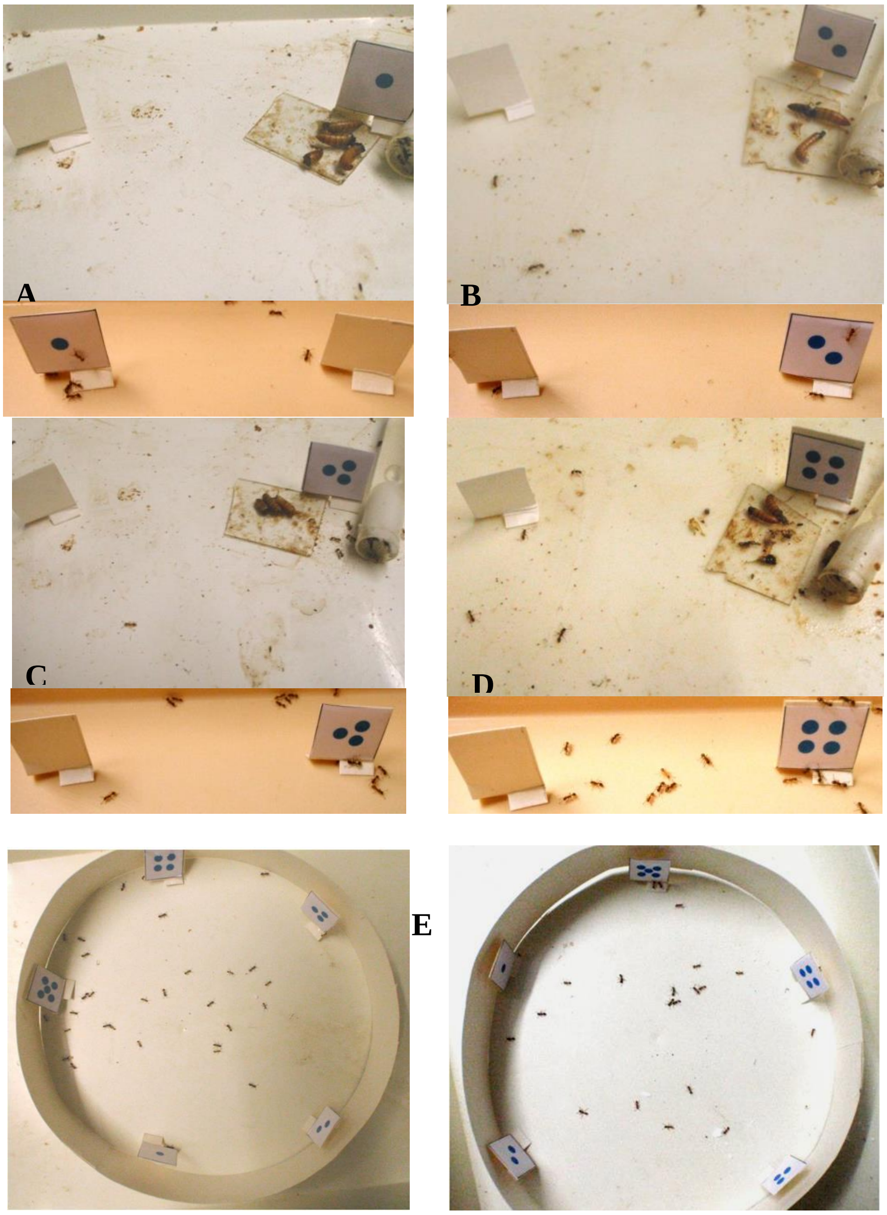

Figure S1. Views of experiments made for examining if ants could anticipate the following number of blue circles in an increasing sequence of 1 to 4 blue circles. **A, B, C, D**: successive training to (upper photos) and testing faced with (lower photos) respectively 1, 2, 3, 4 blue circles *versus* 0 circle. **E**: testing ants of the two colonies in front of 1, 2, 3, 4 and 5 blue circles simultaneous presented in a circular enclosure. They showed a preference for the 5 circles, having thus expected what would be the following number in the increasing sequence.

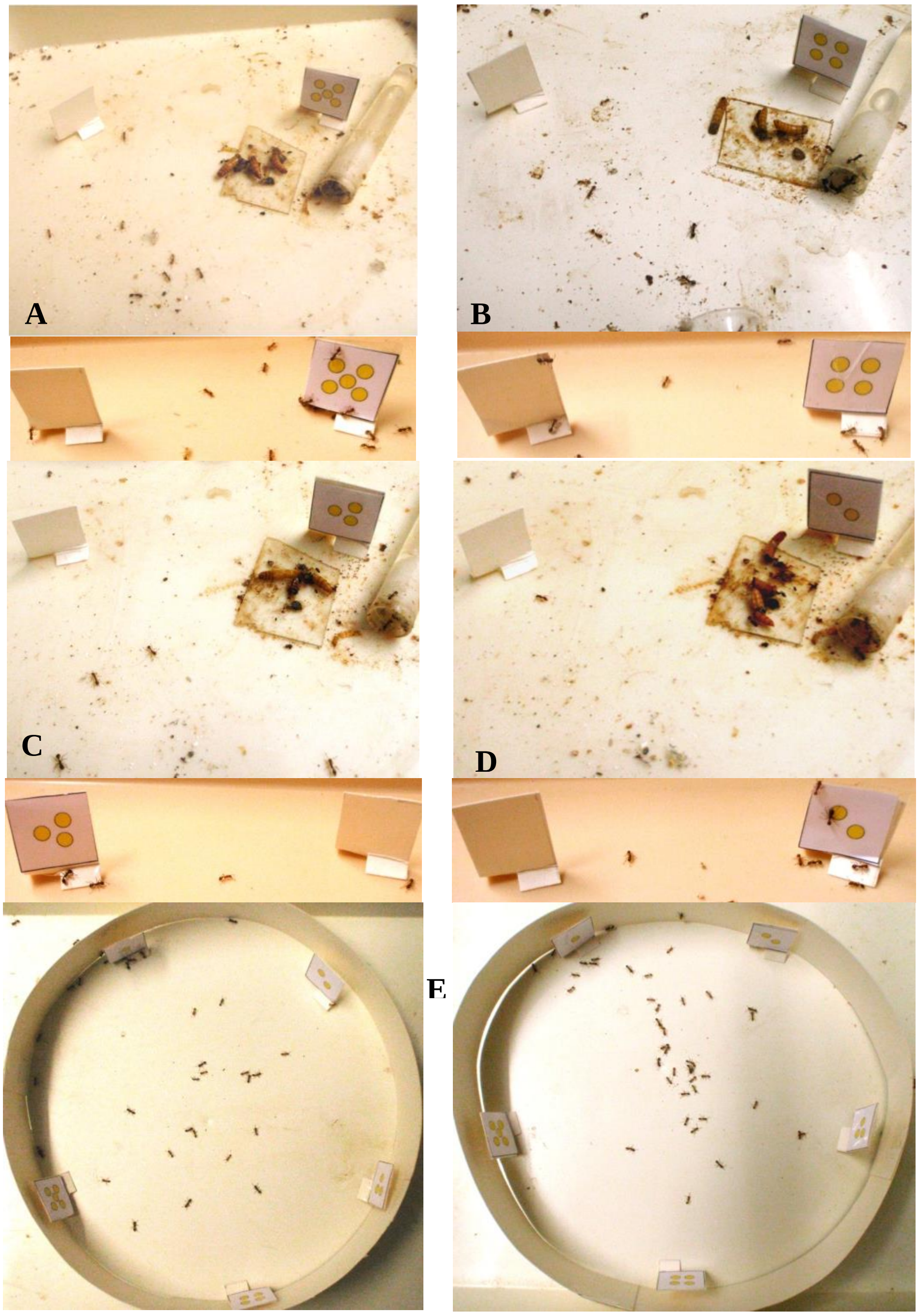

Figure S2. Views of experiments made for examining if ants could anticipate the following number in a decreasing sequence of 5 to 2 yellow circles. **A, B, C, D**: successive training to (upper photo) and testing faced with (lower photo) respectively 5, 4, 3, 2 yellow circles *versus* 0 circle. **E**: testing ants of the two colonies in front of 1, 2, 3, 4 and 5 yellow circles simultaneous presented in a circular enclosure. They went mostly to the 1 circle, having thus expected what would be the following number in the decreasing sequence.
